# An Evaluation of the Implementation of a “No Force First” Informed Organisational Guide to Reduce Physical Restraint in Mental Health and Learning Disability Inpatient Settings in the UK

**DOI:** 10.3389/fpsyt.2022.749615

**Published:** 2022-02-02

**Authors:** Alina Haines-Delmont, Katie Goodall, Joy Duxbury, Anthony Tsang

**Affiliations:** Department of Nursing, Faculty of Health, Psychology and Social Care, Manchester Metropolitan University, Manchester, United Kingdom

**Keywords:** restraint reduction, mental health, learning disabilities, no force first, violence and aggression, inpatient settings

## Abstract

**Background:**

The use of physical restraint on vulnerable people with learning disabilities and mental health problems is one of the most controversial and criticised forms of restrictive practice. This paper reports on the implementation of an organisational approach called “No Force First” within a large mental health organisation in England, UK. The aim was to investigate changes in violence/aggression, harm, and physical restraint following implementation.

**Methods:**

The study used a pretest-posttest quasi-experimental design. Recorded incidents of violence/aggression from 44 inpatient mental health and learning disabilities (including forensic) wards were included (*n* = 13,599). Two study groups were created for comparison: the “intervention” group comprising all incidents on these wards during the 24 months post-implementation (2018–2019) (*n* = 6,551) and the “control” group comprising all incidents in the 24 months preceding implementation (2015–2016) (*n* = 7,048). Incidents recorded during implementation (i.e., 2017) were excluded (*n* = 3,705). Incidence rate ratios (IRR) were calculated with 95% confidence intervals (95% CI). Multivariate regression models using generalised estimating equations were performed to estimate unadjusted and adjusted prevalence ratios (aPR) of physical restraint and harm, using type of wards, incident, and violence/aggression as key covariates.

**Results:**

A significant 17% reduction in incidence of physical restraint was observed [IRR = 0.83, 95% CI 0.77–0.88, *p* < 0.0001]. Significant reductions in rates of harm sustained and aggression/violence were also observed, but not concerning the use of medication during restraint. The prevalence of physical restraint was significantly higher in inpatients on forensic learning disability wards than those on forensic mental health wards both pre- (aPR = 4.26, 95% CI 2.91–6.23) and post-intervention (aPR = 9.09, 95% CI 5.09–16.23), when controlling for type of incident and type of violence/aggression. Physical assault was a significantly more prevalent risk factor of restraint use than other forms of violence/aggression, especially that directed to staff (not to other patients).

**Conclusions:**

This is a key study reporting the positive impact that organisational models and guides such as “No Force First” can have on equipping staff to focus more on primary and secondary prevention as opposed to tertiary coercive practices such as restraint in mental health and learning disabilities settings.

## Introduction

The assumption that conflict in mental health and learning disability settings is inevitable and could only be dealt with by force and physically or medically restraining service users has been challenged for decades in psychiatry (1). The use of physical restraint on people with learning disability and mental health difficulties is the most controversial and debated form of restrictive practice—it has no therapeutic value (2, 3) and it is against people's human rights (4–6). It can traumatise patients, lead to injuries and burnout for staff, frustration and reduced quality of life for carers (3, 7–10) and it can have significant negative economic impact on organisations (11, 12).

There is a major drive in mental health settings to consider the use of restraint as a treatment failure and change focus from containment/coercion to recovery (13, 14). Despite the evidence, lobbying, public policy and guidelines to minimise the use of these controversial practices, there is an indication that these are still commonly used in these settings. Globally, while the frequency of physical restraint on mental health inpatients differs from one country to another and one service to another, ranging from 3.8 to 51.3%, evidence suggests that this has been on the increase in the last decades (15–17). The use of restrictive practices in inpatient settings for people with learning disabilities has generated a lot of criticism and concerns about infringement of human rights and misconduct (18). Recent figures indicate that in England a patient with learning disabilities is restrained, on average, every 15 mins, and the frequency of restraint use has increased over the years (e.g., more than a 50% increase from 2016 to 2017 (19) and more than 70% from 2017 to 2019 (20).

The use of strategies and programmes to minimise the use of restraint has remained a priority both in the UK and internationally. Examples include UK policy documents such as Mental Health Units (Use of Force) Act 2018 (21) and the unanimous decision by the Council of Europe to adopt a resolution to an imminent transition to start eradicating coercive practices in mental health settings (22). There is a wide range of discreet interventions as well as complex programmes or strategies targeted at reducing restrictive practices with the potential to reduce conflict and harm in adult mental health and learning disabilities settings. Examples include the Safewards (23) and the RAID and Positive Behaviour Support (24) approaches developed in the UK and the Engagement model (25), Six Core Strategies (26) and “No Force First” (27) developed in the US.

Developed by Recovery Innovations, Inc., a nonprofit corporation operating a range of recovery-oriented programmes in the US, the “No Force First” model was developed in 2006. The philosophy of the “No Force First” model is that any act of coercion is detrimental to the ultimate recovery of the service user and that a fundamental change in practice and culture can transform an organisation's performance in this area (28). Using force and thus some forms of restrictive practices is incompatible with the values of recovery, such as choice, self-determination, and personhood (29, 30). Practices such as physical and chemical restraint should only be used as a last resort (13, 31–33). Programmes using the “No Force First” model/philosophy seek to transform the experience of service users by minimising and eventually eliminating the use of physical restraint, seclusion and rapid tranquilisation.

Research suggests that “No Force First” informed programmes can be effective in reducing the use of restraint on people with severe psychiatric disorders (27, 34), but more empirical, international studies are needed to strengthen the evidence, covering a wider range of populations, including learning disabilities. Research regarding the impact of this model developed in the US on the use of restrictive practices is limited to a few small-scale studies (14, 34). This study therefore addresses some of the gaps in evidence by reporting results regarding the implementation of a bespoke, person-centred and recovery focused restraint reduction program of interventions in a mental health organisation in the UK.

### Aims and Objectives

The present study aimed to evaluate the impact following the implementation of a “No Force First” informed program of interventions (referred to as “the Guide” hereafter) within inpatient mental health and learning disability settings. These settings included: Adult Mental Health and Psychiatric Intensive Care Units (PICUs), Complex Care (older people), Forensic Mental Health, Learning Disabilities (LD) Services, and Specialist Services (addiction) wards. The specific objectives were to:

1. Investigate the changes in incidences of violence/aggression, changes in duration of physical restraint, number of people involved during a restraint event, type of restraining technique used, medication used during restraint and the method in which it was administered;
2. Examine the overall differences in incidence rates of physical restraint, aggression/violence, harm and medication used during restraint pre- and post-intervention; and
3. Estimate the prevalence of risk factors (i.e., type of violence/aggression, type of incident and type of ward) associated with the use of restraint and harm at population level.

## Materials and Methods

### Study Design and Participants

A pretest-posttest quasi-experimental design was used to examine whether incidents of violence/aggression, harm, and physical restraint were significantly reduced following implementation of the Guide. The posttest (“post-intervention”) group comprising all incidents of patient violence/aggression on these wards during the 24 months post-implementation of the Guide (January 2018—December 2019) was compared with the pretest (“pre-intervention”) group, comprising all incidents in the 24 months preceding implementation (January 2015—December 2016). Incidents recorded while the operationalization of the Guide was ongoing (i.e., January—December 2017) were excluded.

Adult (≥18 years old) male and female patients admitted on the 44 wards who had been involved in at least one incident of aggression/violence during the pretest and posttest periods and who had a formal primary diagnosis of a learning disability or mental health problem were included. Diagnosis was assessed using one of the two established diagnostic frameworks, the ICD-10 (35) or ICD-11 (36), the DSM-IV (37) or DSM-V (38).

### Setting

The organisation provides specialist clinical inpatient and community mental health, learning disabilities, addiction and acquired brain injury services across 80 sites, mainly in the North West of England, serving a population of almost 11 million people. For the purpose of this study, inpatient wards (*n* = 44) covering the following services were included:

- ***Adult Mental Health***: these include gender specific and mixed acute admission wards providing 24 hours assessment and/or treatment for people experiencing mental health difficulties, including adults detained under the Mental Health Act; long stay/rehabilitation focused wards for working age adults; and psychiatric intensive care wards (as below).
- ***Psychiatric Intensive Care Units (PICU)***: wards providing 24 h intensive and specialist care and treatment for service users whose risks and behaviours cannot be managed on an open acute ward.
- ***Complex Care (older people)***: mixed gender acute assessment wards for adults over 65 years, including specialist dementia inpatient wards and services for older people with functional severe and enduring mental health problem.
- ***Forensic Mental Health (high/medium/low secure)***: forensic inpatient services providing care for people detained under the Mental Health Act (39) and subject to different levels of security, depending on the level of assessed danger patients present to others or themselves.
- ***Learning Disabilities (LD) Services*** (low and medium secure units and support teams/ESS): provide treatment for adults (male and female) with a learning disability or other development disorder or autism.
- ***Specialist Services (addiction)***: these wards provide drug and alcohol medically managed detoxification for people who do not meet the criteria for community detox due to comorbid needs or pregnancy.

### The Intervention: An Organisational Guide to Reducing Restrictive Practices

“No Force First” is the approach adopted by a large mental health organisation in the UK with the view to minimise the use of restrictive practices and improve health outcomes. In 2013, this National Health Services (NHS) organisation piloted “No Force First” informed interventions on three wards serving people whose needs included acute adult mental health, learning disability and women forensic medium secure services. The results of the pilot study showed positive results in reducing the use of restraint on these wards, incidents of violence/aggression and staff sickness (14, 18). Following this initial success, the organisation developed a “*Guide to Reducing Restrictive Practice in Mental Health Services*” (40) based on the underpinning philosophy of “No Force First” and focusing on co-production, values-based recruitment, trauma informed care, a recovery ethos, risk sharing partnerships and individualised care. This is achieved using six key bespoke interventions:

- **“*No Force First” engagement sessions***—delivered in partnership with service users, healthcare teams are introduced to “No Force First” and hear accounts of people's experience of physical intervention;
- **“*No Force First” ward criteria and reviewing restrictive practice***—encouraging clinical staff to listen to service users and removing or reducing restrictions and “blanket rules” that can cause frustration and conflict;
- ***Positive handovers***—objective nursing handovers focused on recovery and understanding of past trauma in relation to triggers and behaviours that challenge;
- ***Healthy communities***—giving service users the opportunity to be involved in decision making on how the unit functions, empowering them and giving them a sense of belonging;
- ***Individualised meaningful day***—offering activities that suits service users' individual needs interests and aspirations/fulfilling occupation;
- ***Debriefing for service users and staff***—giving service users and staff the opportunity to reflect on adverse events and identify areas for improvement and learning together.

The tools to support the implementation of these interventions are: the Dynamic Appraisal of Situational Aggression (DASA) (41); Care Zoning (42), One Page Plans, Zonal Observations, HOPE(S) Clinical Model of Care (43), and Safewards interventions (23). These interventions and tools can be used by healthcare teams to reduce conflict and the use of restrictive practices on the wards. While the Guide outlines the “No Force First” philosophy and the tools/interventions, there is flexibility in what healthcare teams use, in line with their population and their needs or setting. For example, the Specialist Learning Disabilities wards have implemented “Safewards” interventions, with “No Force First” as the overarching philosophy and strategy.

### Data and Definitions

Data were extracted from the organisation's official electronic system where all incidents are recorded. Data on the use of restraint were recorded in line with UK reporting requirements (44). The following data were captured:

- Type of service/ward (as above)
- Type of patient violence/aggression (i.e., physical assault; harassment; sexual assault; threatening behaviour; verbal assault; other—including self-harm, hostage taking, play fighting, psychological abuse);
- Direction of violence/aggression (i.e., towards staff or other patients);
- Whether the incident resulted in harm and level of harm sustained (i.e., low/moderate/high);
- Whether the incident resulted in the use of physical restraint, defined by the British Code of practice: Mental Health Act 1983 (2015: 295) as “any direct physical contact where the intervener's intention is to prevent, restrict, or subdue movement of the body, or part of the body of another” (39).
- The position of restraint (i.e., prone, supine, side, standing, seated, kneeling, restrictive escort).
- Number of staff involved in restraint and whether medication was used during restraint;
- The way in which the medication was administered (i.e., by injection/rapid tranquilisation—intramuscular/intravenous; oral; other/nasal spray). It should be noted that PRN (pro re nata) medication would not have been recorded, as it is not officially categorised as a form of restrictive intervention in the UK; and
- Duration of restraint (minutes).

### Outcome Data

The explanatory variables in this study were incidents that were transformed into categorical variables that were divided into three categories:

i. type of wards: forensic mental health, adult mental health, complex care, forensic learning disability (“forensic LD”), specialist support teams learning disability (“specialist LD: ESS) and PICU;
ii. type of incident: physical assault, threatening behaviour, and verbal assault; and
iii. who was the aggression/violence towards (i.e., aggression/violence towards staff and aggression/violence towards patients).

These explanatory variables were used to estimate the prevalence ratios of physical restraint and harm (0 = no presence of outcome, 1 = presence of outcome).

### Analysis

Descriptive statistics (frequencies, crosstabs) were used to report on key characteristics of incidents pre- and post-intervention, such as: type of violence/aggression; proportion of violence/aggression incidents resulting in physical restraint and harm; proportion of restraint incidents resulting in the use of medication and the way the medication was administered; number of staff involved in restraint, position and duration of restraint.

The incidence rates (number of events/per 1,000 patient-days) of physical restraint, violence/aggression, harm sustained and medication used during restraint were calculated for both study periods. The rate difference between the two study periods were calculated using incidence rate ratios (IRRs) with 95% confidence intervals (95% CI).

To account for repeated observations and to provide a population-average interpretation of the results, Poisson generalised estimating equation (GEE) modelling was applied to model longitudinal outcomes in this population (45). An unstructured correlation matrix with robust variance estimator was applied to increase the correctly specified working matrix due to the variability of repeated observations among cases/patients. When this model failed to converge to provide parameter estimates, an exchangeable correlation matrix was applied instead. Bivariate Poisson GEE regression models were performed to estimate the unadjusted prevalence ratio of explanatory variables on physical restraint and harm pre- and post-intervention. Multivariate Poisson GEE regression were performed to estimate unadjusted and adjusted prevalence ratio (aPR) of explanatory variables associated with the two outcomes of interest pre-and post-intervention. Unadjusted and aPR were reported with their respective 95% confidence intervals (95% CI) and *p* values. Patients with missing data or unknown data in any of the variables considered were excluded only from those analyses involving that variable. Moreover, collinearity diagnostics (i.e., tolerance statistics and variance inflation factor) were examined to ascertain multicollinearity amongst the explanatory variables. Evidence of multicollinearity was found (i.e., tolerance statistics were >0.10 or variance inflation statistics were >10) before conducting the GEE regression models, thus some wards were combined with the highly correlated wards to improve statistical power of estimation of prevalence ratios, e.g., “complex care (older people)” wards were combined with “specialist services (addiction)” wards. All analyses were performed using SPSS version 26 (46).

## Results

### Patients' Characteristics

The majority of patients admitted on the study wards during the time of investigation were male (*n* = 5,606, 63.1%), of a White ethnic background (*n* = 7,337, 82.6%) and reported to be Christian (*n* = 5,257, 59.1%). Just under two thirds of inpatients were comprised of young people aged 18–47 (*n* = 5,527, 62.2%), a quarter middle aged (48–63) (*n* = 2,152, 24.2%) and *n* = 1,191 (13.4%) elderly (64+ years old). A high proportion of participants disclosed being heterosexual (*n* = 5,134, 57.8%), but there is a significant amount of information missing or not being disclosed regarding the sexual orientation of participants (*n* = 3,337, 37.5%). For additional patient characteristics, please refer to Table 1.

**Table 1:** Patients' demographics.

	**Pre-intervention (***n***, %)**	**Post-intervention (***n***, %)**	**Population (intervention wards)**
	4,684	4,199	8,883
Age			
Young (18–47)	2,981 (63.9)	2,546 (60.7)	5,527 (62.2)
Middle Age (48–63)	1,085 (23.2)	1,067 (25.4)	2,152 (24.2)
Elderly (64+)	607 (13.0)	584 (13.9)	1,191 (13.4)
Missing/other*	10	2	12 (0.13)
Gender			
Female	1,735 (37)	1,542 (36.7)	3,277 (36.9)
Male	2,949 (63)	2,657 (63.3)	5,606 (63.1)
Ethnicity			
Asian/Asian British	84 (1.8)	46 (1.1)	130 (1.46)
Black/African/Caribbean/Black British	134 (2.8)	134 (3.2)	268 (3.0)
Mixed/multiple	92 (2)	98 (3.2)	190 (2.1)
Other	35 (0.74)	63 (1.5)	98 (1.1)
White	3,975 (84.8)	3,362 (80)	7,337 (82.6)
Not reported/disclosed/missing	364 (7.8)	484 (11.5)	848 (9.54)
Religion			
Christian	3,120 (66.6)	2,137 (50.9)	5,257 (59.1)
Muslim	104 (2.2)	85 (2.02)	189 (2.12)
Jewish	12 (0.25)	7 (0.16)	19 (0.21)
Hindus	22 (0.47)	12 (0.28)	34 (0.38)
Buddhist	19 (0.40)	23 (0.54)	42 (0.47)
Atheist/non believer/not attached	865 (18.4)	660 (15.7)	1,525 (17.1)
Other	28 (0.6)	26 (0.6)	54 (0.60)
Not reported/disclosed/missing	510 (10.8)	1,241 (29.5)	1,751 (19.7)
Sexual orientation			
Bisexual	65 (1.38)	50 (1.2)	115 (1.3)
Gay and lesbian	85 (1.81)	26 (0.61)	111 (1.25)
Heterosexual	3,833 (81.8)	1,301 (31)	5,134 (57.8)
Not reported/disclosed/missing	541 (11.5)	2,796 (66.5)	3,337 (37.56)

* *Twelve patients were under the age of 18 at the time of admission*.

A total of 2,038 inpatients were included in the regression analyses: pretest (*n* = 969) and posttest (*n* = 1,069).

### Frequency, Characteristics, and Outcomes of Incidents of Violence/Aggression

There were 7,048 incidents of patient to staff or patient to patient violence/aggression incidents pre-intervention (2015–2016) and 6,551 post-intervention (2018–2019), with a total of 13,599 incidents recorded on the 44 wards. Incidents recorded while the operationalization of the Guide was ongoing were excluded (*n* = 3,705). The most frequent types of incident recorded in the pre-intervention period were physical assault (*n* = 2,772, 39.4%), threatening behaviour (*n* = 2,573, 36.5%) and verbal assault (*n* = 1,617, 23.0%). Post-intervention, incidents of threatening behaviour were slightly more frequent (*n* = 2,677, 40.9%) than physical assault (*n* = 2,561, 39.1%), and there was a decrease in incidents of verbal assault (*n* = 1,236, 18.9%).

As shown in Table 2 below, most incidents were recorded on forensic mental health wards (*n* = 5,717, 42%) during both pre- and post-intervention period, while adult mental health wards experienced the least number of incidents (*n* = 425, 3%). Majority of violence or aggression was directed to staff rather than patients in all types of services. There was a reduction in frequency of incidents of violence/aggression for all types of services, a part from PICU.

**Table 2:** Incidents of violence/aggression pre- and post-intervention by type of service/ward.

	**Violence/aggression towards staff** ***n*** **(%)**		**Violence/aggression towards other patients** ***n*** **(%)**		**Violence/aggression** ***n*** **(%)**		
	**PRE**	**POST**	**PRE**	**POST**	**PRE**	**POST**	**TOTAL**
Forensic mental health	2,646 (88.1)	2,413 (88.9)	356 (11.9)	302 (11.1)	3,002 (42.6)	2,715 (41.4)	5,717 (42.0)
Adult mental health	231 (79.4)	107 (79.9)	60 (20.6)	27 (20.1)	291 (4.1)	134 (2.0)	425 (3.1)
Complex care (older people) and specialist services (addiction)	1,123 (64.9)	1,354 (67.5)	607 (35.1)	652 (32.5)	1,730 (24.5)	2,006 (30.6)	3,736 (27.4)
Learning disabilities services (incl. forensic and specialist/ESS)	1,309 (76.4)	1,031 (78.5)	404 (23.6)	283 (21.5)	1,715 (24.3)	1,338 (20.4)	3,053 (22.4)
PICU	202 (65.2)	273 (76.3)	108 (34.8)	85 (23.7)	310 (4.4)	358 (5.5)	668 (4.9)
Total	5,511* (78.2)	5,178* (79.3)	1,535* (21.8)	1,349* (20.7)	7,048 (100)	6,551 (100)	13,599 (100)

* *Missing data for 2 incidents for pre- and 24 incidents for post-intervention regarding direction of violence/aggression (towards staff or patients)*.

Just over a quarter of incidents of violence/aggression resulted in harm (*n* = 1,807, 25.6%) pre-intervention and this decreased to 21.8% (*n* = 1,428) post-intervention. The highest percentage of incidents of harm were classed as “low-level harm” for both pre- (*n* = 1,637, 90.6%) and post-intervention (*n* = 1,277, 89.4%). High level harm was recorded for a very small proportion of incidents for both periods under investigation (*n* = 6, 0.33% and *n* = 2, 0.14%, respectively).

Information on whether an incident of violence/aggression resulted in the use of physical restraint was missing for a large number of cases both pre- (*n* = 1,258, 17.8%) and post-intervention (*n* = 1,658, 25.3%). Recorded data on the use of physical restraint indicated that two thirds of incidents resulted in restraint pre-intervention and just under a third post-intervention (n = 1,890, 67.4% and *n* = 1,538, 31.4% respectively). In terms of restraining techniques, a standing position was most frequently recorded both pre- and post-intervention, followed by supine position. A prone position was used in 6.9% of restraint events (*n* = 129) pre-intervention and this decreased to 4.7% (*n* = 68) post-intervention. Two to three number of staff were most frequently involved in restraint for a duration of up to 5 mins, and in a small proportion of cases additional medication/rapid tranquilisation was used, although there were significant missing data for these variables. For a breakdown of results regarding characteristics of restraint see Supplementary Material.

### Incidence Rate Ratios for Violence/Aggression, Physical Restraint and Harm

There were 456,487 patient days and 22,932 admissions during the pre-intervention period (January 2015—December 2016) and 449,827 patient-days and 21,062 admissions during post-intervention (January 2018—December 2019). Table 3 shows the number of events and incidence rates of physical restraint, aggression/violence, harm sustained, and medication used during restraint for both study periods. The overall incidence rate significantly decreased by 20% in patients who sustained harm when comparing the two study periods (IRR = 0.80, 95% CI 0.74–0.87, *p* < 0.0001). A lower, but still statistically significant reduction in incidence rates was found for aggression/violence (IRR = 0.94, 95% CI 0.91–0.97, *p* < 0.0001) and physical restraint outcomes (IRR = 0.83, 95% CI 0.77–0.88, *p* < 0.0001). Although there was a 11% decrease in the use of medication during restraint, this was found to be statistically insignificant (IRR = 0.89, 95% CI 0.79–1.00, *p* = 0.06).

**Table 3:** Incidence rates and number of events of physical restraint, aggression/violence, harm sustained, medication used during restraint.

	**Number of events**		**Incidence rate (per 1,000 patient-days)**			
	**Pre-intervention**	**Post-intervention**	**Pre-intervention**	**95% CI**	**Post-intervention**	**95% CI**
Physical restraint	1,890	1,538	4.14	3.96–4.33	3.42	3.25–3.59
Aggression/violence	7,046	6,527	15.44	15.08–15.80	14.51	14.16–14.87
Harm sustained	1,807	1,428	3.96	3.78–4.14	3.17	3.01–3.34
Medication used during restraint	681	597	1.49	1.38–1.61	1.33	1.22–1.44

### Prevalence of Risk Factors Associated With Physical Restraint

Table 4 displays the bivariate and multivariate analyses of the factors associated with the prevalence of physical restraint pre- and post-intervention of the study. All explanatory variables were significantly associated with physical restraint pre-intervention except for complex care (PR = 1.76, 95% CI 1.01–3.06, *p* = 0.05). During pre-intervention, the most significant prevalent risk factor of physical restraint when controlling for covariates were patients being in forensic learning disability wards (aPR = 4.26, 95% CI 2.91–6.23, *p* < 0.0001) followed by PICU (aPR = 3.41, 95% CI 2.44–4.75, *p* < 0.0001) when compared to forensic mental health wards. Additionally, the prevalence of physical restraint was significantly lower in threatening behaviour (aPR = 0.66, 95% CI 0.49–0.88, *p* = 0.005) and verbal assault (aPR = 0.41, 95% CI 0.24–0.69, *p* < 0.0001) compared with physical assault. During post-intervention, patients being in forensic learning disability and specialist LD: ESS wards were 8.44 (95% 6.72–10.61, *p* < 0.0001) and 8.21 (95% CI 6.39–10.54, *p* < 0.0001) fold more prevalent than those in forensic mental health to be physically restrained. The prevalence of physical restraint when controlling for other covariates was highest in patients in forensic learning disability wards (aPR = 9.09, 95% CI 5.09–16.23, *p* < 0.0001), followed by specialist learning disability wards (ESS) (aPR = 8.25, 95% CI 4.67–14.56, *p* < 0.0001) and PICU (aPR = 7.30, 95% CI 3.63–14.69, *p* < 0.0001) when compared to forensic mental health wards. Whereas the prevalence of physical restraint was significantly lower in patients who engaged in verbal assault (aPR = 0.08, 95% CI 0.04–0.18, *p* < 0.0001) and behaved aggressively/violently towards other patients (aPR = 0.70, 95% CI 0.60–0.81, *p* < 0.0001) when compared to those who engaged in physical assault and behaved aggressively towards staff, respectively.

**Table 4:** Prevalence ratio associated with physical restraint.

	**Pre-intervention 2015–2016** **(*****N*** **=** **969)**		**Post-intervention 2018–2019** **(***N*** = 1,069)**	
	**Unadjusted prevalence ratios** **(95% CI)**	**Adjusted prevalence ratios** **(95% CI)**	**Unadjusted prevalence ratios** **(95% CI)**	**Adjusted prevalence ratios** **(95% CI)**
**Type of wards**				
Forensic mental health^a^				
Adult mental health	1.89 (1.10–3.24)*	1.88 (1.35–2.60)***	2.61 (1.20–3.41)^b***^	0.52 (2.12–9.72)***
Complex care (older people)	1.76 (1.011–3.06)	1.50 (1.04–2.16)*	2.15 (1.58–2.92)^b***^	3.53 (1.73–7.22)***
Forensic learning disability	5.88 (3.49–9.92)***	4.26 (2.91–6.23)***	8.44 (6.72–10.61)^b***^	9.09 (5.09–16.23)***
Other learning disability (Specialist: ESS)	3.03 (1.68–5.47)***	2.42 (1.63–3.59)***	8.21 (6.39–10.54)^b***^	8.25 (4.67–14.56)***
PICU	3.76 (2.17–6.50)***	3.41 (2.44–4.75)***	4.37 (3.12–6.11)^b***^	7.30 (3.63–14.69)***
**Type of incident**				
Physical assault^a^				
Threatening behaviour	0.47 (0.39–0.58)***	0.66 (0.49–0.88)**	0.48 (0.41–0.56)***	0.52 (0.25–1.09)
Verbal assault	0.32 (0.19–0.53)***	0.41 (0.24–0.69)**	0.17 (0.14–0.20)***	0.08 (0.04–0.18)***
**Who was the aggression/violence towards?**				
Towards staff^a^				
Towards patient	0.66 (0.58–0.75)***	0.58 (0.50–0.68)***	0.70 (0.62–0.80)***	0.70 (0.60–0.81)***

a *Indicates the reference category*.

b *Analyses were conducted using an exchangeable correlation matrix*.

* *p < 0.05*;

** *p < 0.01*;

*** *p < 0.001*.

### Prevalence of Risk Factors Associated With Harm

Table 5 displays the bivariate and multivariate analyses of the factors associated with the prevalence of harm pre- and post-intervention of the study. The estimated adjusted prevalence ratios during pre-intervention indicated that harm was significantly more prevalent in forensic disability wards (aPR = 7.24, 95% CI 2.43–21.57, *p* < 0.0001) compared to those in forensic mental health wards. Incidents of threatening behaviour (aPR = 0.48, 95% CI 0.34–0.68, *p* < 0.0001) and verbal assault (aPR = 0.67, 95% CI 0.55–0.81, *p* < 0.0001) were both significantly associated with a lower incidence of being harmed when compared to incidents that involved physical assault. Harm was significantly more prevalent in incidents of aggression/violence shown towards other patients (aPR = 1.26, 95% CI 1.01–1.57, *p* = 0.04) when compared to incidents of aggression/violence shown towards staff. Post-intervention multivariate analyses showed that all explanatory variables were significantly associated with patients being harmed. The prevalence of harm towards patients were highest in forensic learning disability (aPR = 38.48, 95% CI 25.01–59.20, *p* < 0.0001) and specialist learning disability (ESS) wards (aPR = 32.24, 95% CI 20.47–50.76, *p* < 0.0001) followed by adult mental health (aPR = 2.48, 95% CI 1.55–3.91, *p* < 0.0001) and PICU (aPR = 2.31, 95% CI 1.23–4.33, *p* = 0.01) when compared to patients in forensic mental health. Threatening behaviour (aPR = 0.59, 95% CI 0.48–0.72, *p* < 0.0001) was the least prevalent type of incident associated with harm when compared to physical assault. The prevalence of harm increased and remained significant in incidences of aggression/violence towards other patients (aPR = 1.47, 95% CI 1.33–1.61, *p* < 0.0001) when compared to incidences of aggression towards staff, even after controlling for other covariates.

**Table 5:** Prevalence ratio associated with harm.

	**Pre-intervention 2015–2016** **(*****N*** **= 969)**		**Post-intervention 2018–2019** **(*****N*** **= 1,069)**	
	**Unadjusted prevalence ratios** **(95% CI)**	**Adjusted prevalence ratios** **(95% CI)**	**Unadjusted prevalence ratios** **(95% CI)**	**Adjusted prevalence ratios** **(95% CI)**
**Type of wards**				
Forensic mental health^a^				
Adult mental health	2.72 (1.95–3.77)^b***^	0.84 (0.31–2.29)	3.55 (2.21–5.71)^b***^	2.48 (1.55–3.97)^b***^
Complex care (older people)	2.35 (1.54–3.59)^b***^	0.68 (0.23–2.00)	3.38 (1.98–5.75)^b***^	2.28 (1.34–3.88)^b***^
Forensic learning disability	28.30 (21.63–37.03)^b***^	7.24 (2.43–21.57)***	64.03 (42.77–95.87)^b***^	38.48 (25.01–59.20)^b***^
Other LD (Specialist/ ESS)	1.73 (0.80–3.76)^b^	0.46 (0.13–1.70)	55.36 (36.45–84.06)^b***^	32.24 (20.47–50.76)^b***^
PICU	4.09 (2.79–6.01)^b***^	1.12 (0.40–3.25)	3.31 (1.74–6.31)^b***^	2.31 (1.23–4.33)^b**^
**Type of incident**				
Physical assault^a^				
Threatening behaviour	0.38 (0.31–0.47)***	0.48 (0.34–0.68)***	0.20 (0.11–0.35)***	0.59 (0.48–0.72)^b***^
Verbal assault	0.59 (0.44–0.80)***	0.67 (0.55–0.81)***	0.23 (0.11–0.50)***	0.71 (0.61–0.82)^b***^
**Who was the aggression/violence towards?**				
Towards staff^a^				
Towards patient	1.28 (0.89–1.86)	1.26 (1.01–1.57)*	1.63 (1.30–2.04)***	1.47 (1.33–1.61)^b***^

a *Indicates the reference category*.

b *Analyses were conducted using an exchangeable correlation matrix*.

* *p < 0.05*;

** *p < 0.01*;

*** *p < 0.001*.

## Discussion

### Key Findings

This paper reports positive results regarding the implementation of a bespoke, person-centred and recovery focused “No Force First” intervention in a 'large mental health organisation in England, UK. In particular, a notable reduction in the use of restraint was found. The reduction of restrictive practices and containment such as restraint using organisational models of this sort has been reported elsewhere, both in the UK and globally. The Six Core Strategies for example have a strong and growing evidence base highlighting their impact upon restraint and seclusion reduction (9, 47, 63). The UK had also reported reductions in the use of physical restraint following the implementation of an adapted version of the 6CS called REsTRAIN Yourself (48) and similar positive results have been evidenced in a Finnish RCT (49). The positive results showed a reduction in the types of restraint used and their negative impact.

With regards to the type of aggression, the results indicate that incidents of violence/aggression were most frequent within forensic mental health settings. This could be due to most inpatients in these settings being male and/or involuntarily detained, which has been shown to be linked to higher rates of inpatient violence (50). Previous research indicates that, while incidence of violence is linked to ~20% of inpatients on (acute) adult mental health wards (50), these rates double for forensic mental health wards (51). Incidents ranged from verbal abuse and threatening behaviour to physical assault. Physical assault was found to be the most common type of violence/aggression on the study wards pre-intervention (39.4%), but this was reduced post-intervention, when the most frequent type of violence/aggression was threatening behaviour (40.9%). This is in comparison to research in the UK on acute mental health wards, where verbal aggression has been the most common type of aggression reported (51%) (52). The finding that the majority of incidents of violence/aggression were directed towards staff rather than patients is in keeping with the general literature irrespective of setting or type of incident (31, 53–55). When one looks at the consequences of violence/aggression around a quarter of incidents resulted in harm, mainly minor, implying that the incident required extra observation or minor treatment for one or more persons.

With regards to coercive practices, the proportion of incidents resulting in the use of restraint was on average 50% but decreased from 67.4% pre-intervention to 31.4% post-intervention. It is difficult to compare these results with previous research, given the variation in use of coercive measures and the way incidence of the use of physical restraint is measured. However, some evidence suggests that while approximately half of aggressive incidents result in the use of seclusion, only a small proportion target the use of restraint (56, 57). In contrast, other studies indicate that a higher proportion of incidents result in the use of manual or medical restraint (58–60) and only a fifth of incidents of aggressive behaviour are subject to seclusion (60–62). Previous research using “No Force First” informed interventions mirrors these positive results (34).

Results demonstrate that a standing position was the most frequently method of restraint throughout the duration of the study. This may be a reflection of the current trend to minimise and, some would argue eradicate, the use of prone restraint given concerns over its safety and subsequent changes in policy to avoid its use (31, 33, 64–66). Whilst prone restraint was still reported in this study, its use reduced from 6.9% pre-intervention to 4.7% post-intervention.

The prevalence of physical restraint and harm was significantly higher in inpatients on forensic learning disability wards than those on forensic mental health wards both pre- and post-intervention. Physical assault was significantly more of a prevalent risk factor of restraint use than other forms of violence/aggression, when this was directed to staff, and more of a risk factor of being harmed when the violence/aggression was directed to other patients. This is in line with previous research showing that aggression against others is strongly associated with restrictive practices, including restraint and seclusion (16, 67). The characteristics, culture and details of incidents can clearly play a role in whether a patient is restrained or there is harm as a result of violence/aggression. The use of debriefing has been promoted for some time now and is clearly an important tool in understanding the individual and organisational nature of and response to conflict in mental health settings (68).

Fundamentally, this study shows the positive impact that organisational models and guides such as “No Force First” can have on equipping staff to focus more on primary and secondary prevention as opposed to tertiary coercive practices such as restraint. We have been able to report upon that a significant 17% reduction in incidence of physical restraint following the introduction of “No Force First” in addition to reductions in associated rates of harm sustained and episodes of aggression and violence.

### Strengths and Limitations

This is one of the few studies investigating the potential impact of a “No Force First” informed intervention in both mental health and learning disabilities settings. Current evidence was based on two studies: i) a small scale descriptive study conducted in one crisis centre for people with severe psychiatric disorders exploring results to do with the use of chemical restraint (34); and ii) a UK-based service evaluation (14). As data from an entire mental health organisation were included, this study has more power over those focusing on a single ward or unit. It is also assumed that seasonal or trend influences were eliminated given the data were collected over a four-year period. The strength of this study is the large sample size and number of incidents, including the use of GEE models for data analysis. GEE models are more robust and resilient to model misspecification and inferences about the population-average rather than individual-average can be made (69). To our knowledge, this type of analysis has not been reported previously on incidents of violence/aggression, physical restraint and harm in both mental health and learning disabilities settings.

Several caveats should be considered when interpreting the findings. Firstly, the under-reporting of both incidents of violence/aggression and the use of restrictive practices, despite national guidelines and requirements to report all incidents of physical and chemical restraint. It should also be noted that socio-demographics data for the patients could not be linked to the incident data. Therefore, these were not included in the GEE regression models. The lack of explanatory variables available and the variability of repeated measurements among the patients may have attributed to the wide confidence intervals in some of the findings.

Another limitation is linked to intervention fidelity. It was unknown to the researchers the precise timing of the implementation of the intervention and the extent to which components of the intervention were introduced, especially as healthcare teams had the flexibility to choose the most appropriate interventions for their ward/population. To minimise potential “contamination” between the two cohorts, data for the year in which the intervention was in the process of being implemented across the 44 study wards were excluded. This does not imply, however, that components of the intervention were not introduced at all during the pre-intervention period.

Finally, the absence of a control group in the study impacts on the internal validity and potentially reduces the robustness of our findings. However, this study pragmatically evaluated the implications of the Guide implemented by ward staff in real-world settings. There are ethical and practical challenges in mental health and learning disability settings with regards to patients' access to interventions aimed to reduce the instances of restrictive practices and thus impacting on their health, well-being and chances of recovery (especially if this access were denied). The pre-post design was thought to be more appropriate given these challenges. It is worth noting that no significant political, organisational and legal changes have been observed during the study period, a part from the implementation of the Guide within the entire organisation, which was the subject of our investigation.

### Implications for Research and Practice

Further research should focus on investigating the way in which the intervention works in each setting and the degree to which key components of the intervention contribute to a reduction in restraint, violence or harm. It would also be useful to further investigate other characteristics that can determine whether incidents of violence/aggression on the wards are followed by physical restraint (with or without the use of medication) in both mental health and learning disabilities settings, for example patient and staff demographics, staff turnover and burnout, physical environment characteristics, or ward climate. A qualitative study exploring staff's decision to use restraint would also be useful to improve understanding regarding the decision-making process and support the development of preventative strategies.

Healthcare organisations should be more proactive and systematic in their data collection to enable such explorations, but also to support their quality improvement processes. Data on both physical and psychological outcomes following the use of restraint, including patient trauma and service satisfaction, staff post-traumatic stress and absenteeism, job satisfaction should be collected. This will enable a more comprehensive data informed strategy for the prevention and management of conflict in these settings.

Given that the most prevalent predictor of restraint was the nature/type of violence/aggression, healthcare teams should concentrate on both de-escalation during incidents and interventions that prevent violence/aggression from arising in the first place. Additionally, special attention should be paid to learning disabilities settings, as this research points to a significantly higher prevalence of physical restraint and harm in inpatients on forensic learning disability wards compared to forensic mental health wards.

## Conclusion

This study addresses a gap in evidence regarding “No Force First” recovery focused interventions used to reduce the use of restraint in both mental health and learning disabilities settings. The successful translation and impact of a US approach to the UK is of key importance in addressing the reduction of restraint across international settings. Nationally and internationally, it is widely recognised that, in line with “No Force First” philosophy, restrictive practices should be the last option on a list of potential approaches or interventions to deal with distress or potentially threatening behaviour (13, 33). Services are encouraged to move away from coercion and containment towards a more recovery focused care (28). As stated by the developers of “No Force First”, “Force must be the last response considered, and its use implies a treatment failure” and “… the highest price of all is the price paid by the people who are restrained: recovery is stalled by a practice that can disempower them, break their spirit, and reignite a sense of helplessness” (34): 417. While there are still questions and concerns regarding the controversial use of restrictive practices, the implementation of recovery-based models targeted at reducing restraint show promising results.

## Data Availability Statement

The raw data supporting the conclusions of this article will be made available by the authors, without undue reservation.

## Ethics Statement

The studies involving human participants were reviewed and approved by Health Research Authority (HRA), reference number: IRAS 277787. Written informed consent for participation was not required for this study in accordance with the national legislation and the institutional requirements.

## Author Contributions

AH-D secured the funding for the study, was the principal investigator for the study, negotiated access to data, and drafted the manuscript. KG secured the ethical and governance approvals for the study, negotiated access to and collected data, and critically reviewed the manuscript. JD critically reviewed the manuscript. AT designed and wrote the statistical analysis plan, conducted the incidence rate ratio analyses and generalised estimating equations modelling, interpreted the results, participated in manuscript writing and critically reviewed all sections of the manuscript.

## Funding

This work was supported by Mersey Care NHS Foundation Trust (Manchester Metropolitan University WorkTribe ref 230649).

## Conflict of Interest

The authors declare that the research was conducted in the absence of any commercial or financial relationships that could be construed as a potential conflict of interest.

## Publisher's Note

All claims expressed in this article are solely those of the authors and do not necessarily represent those of their affiliated organizations, or those of the publisher, the editors and the reviewers. Any product that may be evaluated in this article, or claim that may be made by its manufacturer, is not guaranteed or endorsed by the publisher.
