# Supplementary material for: An Evaluation of the Implementation of a “No Force First” Informed Organisational Guide to Reduce Physical Restraint in Mental Health and Learning Disability Inpatient Settings in the UK
Source: Front Psychiatry. 2022 Feb 2;13:749615. doi: 10.3389/fpsyt.2022.749615 (PMC8851567; doi:10.3389/fpsyt.2022.749615)
Supplement: Supplementary file 1 [file Table_1.docx]

# Supplementary Material

***Supplementary Table. Characteristics of physical restraint pre- and post-intervention***

|  |  | **PRE** |  | **POST** |  |
| --- | --- | --- | --- | --- | --- |
| **Position** |  | **n** | **%** | **n** | **%** |
|  | Kneeling | 27 | 1.4 | 9 | .6 |
|  | Standing | 656 | 35.1 | 464 | 32.0 |
|  | Prone | 129 | 6.9 | 68 | 4.7 |
|  | Supine | 514 | 27.5 | 414 | 28.6 |
|  | Restricted escort | 153 | 8.2 | 101 | 7.0 |
|  | Seated | 356 | 19.1 | 351 | 24.2 |
|  | Side | 32 | 1.7 | 43 | 3.0 |
|  | Total | 1867 | 100.0 | 1450 | 100.0 |
| **Number of staff involved** |  |  |  |  |  |
|  | 1 | 21 | .7 | 26 | .8 |
|  | 2 | 614 | 19.5 | 461 | 14.4 |
|  | 3 | 451 | 14.3 | 263 | 8.2 |
|  | 4 | 333 | 10.6 | 209 | 6.5 |
|  | 5 | 200 | 6.4 | 155 | 4.8 |
|  | More than 5 | 168 | 5.3 | 129 | 4.0 |
|  | Unknown | 1361 | 43.2 | 1953 | 61.1 |
|  | Total | 3148 | 100.0 | 3196 | 100.0 |
| **Medication** |  |  |  |  |  |
|  | No | 4315 | 61.2 | 3504 | 53.5 |
|  | Unknown | 2052 | 29.1 | 2450 | 37.4 |
|  | Yes | 681 | 9.7 | 597 | 9.1 |
|  | Total | 7048 | 100.0 | 6551 | 100.0 |
| **Administration of medication** |  |  |  |  |  |
|  | Intramuscular (IM) | 211 | 30.9 | 139 | 23.1 |
|  | Intravenous (IV) | 3 | 0.4 | 3 | 0.5 |
|  | Nasal | 1 | 0.1 | 251 | 41.9 |
|  | Oral | 438 | 64.3 | 0 | 0 |
|  | Unknown | 28 | 4.1 | 206 | 34.3 |
|  | Total | 681 | 100.0 | 599 | 100.0 |
| **Duration of restraint** |  |  |  |  |  |
|  | 0-5 minutes | 145 | 94.7 | 211 | 97.2 |
|  | 5-10 minutes | 4 | 2.6 | 2 | 0.9 |
|  | 10-15 minutes | 0 | 0 | 1 | 0.4 |
|  | 15-20 minutes | 2 | 1.3 | 1 | 0.4 |
|  | over 25 minutes | 2 | 1.3 | 2 | 0.9 |
|  | Total | 153 | 100.0 | 217 | 100.0 |
